# Supplementary material for: Chromothripsis during telomere crisis is independent of NHEJ, and consistent with a replicative origin
Source: Genome Res. 2019 May;29(5):737–49. doi: 10.1101/gr.240705.118 (PMC6499312; doi:10.1101/gr.240705.118)
Supplement: Supplemental Material [file supp_gr.240705.118_Supplemental_file_1.zip › contigs/annotated_contigs/DB53/contig.2.DB53_length_752_mean_cov_8.12234042553.docx]

**DB53_length_752_mean_cov_8.12234042553**

```
TGCAGCTGTGGCCCTGATCAGCCAGCGAAGCTCACATTTATTCAGTATGTAAGTGACAAAAGTCTTGAGTAAACACCACTAGAGGGTAA
 >chr21:23746865-23747174 - E=4e-174
TTGACATTGCCGACACCCCCCTCCTCCCGCGCCCCAGTAGAGAGCAATTATGCACCGGTGGGAAATCAAAGGTTGGTCTTAGGACCACA

TGAGTAAACAAGCTATTTAGATAAACTCCCTTGAATTCCTTTGTTATTTACCCTTGCTATTAGCTCAAAGAGGATTAGGCTGCTTCAGC

AGTATCTTTTACTGAAGCTATGCAAAACCCCGGCCTTCCTGG|CTGGGGT|CAAGCTGTCGATGAATCCATCTTTCTGGGTGGGGTCTG
                                                  >chr21:23745171-23745607 - E=2e-250
GAGGACTGTGGCCCAATTCCCACAATTCCACTAGTCAGTGCTTCAGTGGGGACCCTGTGTGGGGGCTGCAACCCCACATTTCTCCTTTG

CATTCATCGACAGCTTGGAAGGCACTCAGTGCTAGCCCATGAAAGCAACTGTAGAGACTGTACCCTGCAAAATCAGATGTAAGGCATAT

CTAGTGAGGATTAATTCTTTTTTATTTTATTTGGAAAGTCCTTATCTCTTCATTTTTGATGAATAAGTTTTCTCATGTTCATGAGTTGA

AAGCCTTATTACTGTTAAAATGTCCATACTACCTAAAGTGATCTAAAGTCCCAGTGTAATCTCTACCAAAATCTTGATTTTTTAATATA

GAAAGAGAAAGAAGTTCCAAAATTTATGTGAAACTGCAAAAG
```
